# Supplementary material for: Sevoflurane Induces a Cyclophilin D-Dependent Decrease of Neural Progenitor Cells Migration
Source: Int J Mol Sci. 2023 Apr 4;24(7):6746. doi: 10.3390/ijms24076746 (PMC10095407; doi:10.3390/ijms24076746)
Supplement: Supplementary file 1 [file ijms-24-06746-s001.zip › ijms-2293014-supplementary.pdf]

Table S1. Proteomics comprehensive results following sevoflurane treatment.

Term	PValue	FDR(false disc	Genes	Pop Hits	Pop Total	Fold Enrichme	Bonferroni	Benjamini	List Total	Count	%
GO:0008137~NADH dehydrogenase (ubiquinone) activity	3.80E-37	4.75E-34	P19404, O43676, P49821, O75489, Q9P0J0, O96000, P51970, O00483, O75438, O95139, P56556, O43678, O43181, P17568, O95299, Q9UI09, Q16718, Q9Y6M9, O75251, O95167, O75380, Q16795, O95182, O75306	48	16881	66.46062992	7.45E-35	7.45E-35	127	24	18.75
GO:0046933~proton-transporting ATP synthase activity, rotational mechanism	1.24E-15	1.52E-12	P24539, P06576, P48201, O75964, P48047, P30049, P25705, O75947, P36542, P56381	17	16881	78.18897638	2.39E-13	1.20E-13	127	10	7.8125
GO:0003755~peptidyl-prolyl cis-trans isomerase activity	2.75E-14	3.44E-11	P62937, Q9Y237, Q9NYL4, Q14318, P23284, Q15257, Q02790, P62942, P30405, P26885, Q08752, Q00688	46	16881	34.67511126	5.40E-12	1.80E-12	127	12	9.375
GO:0022857~transmembrane transporter activity	2.40E-12	3.00E-09	P24539, P06576, O75964, P48047, P30049, P18859, P25705, P56385, O75947, P36542, P56381	49	16881	29.8394665	4.70E-10	1.18E-10	127	11	8.59375
GO:0003954~NADH dehydrogenase activity	4.24E-12	5.30E-09	P49821, O75489, Q9P0J0, O75438, O75251, Q16795, O75306	8	16881	116.3061024	8.31E-10	1.66E-10	127	7	5.46875
GO:0004722~protein serine/threonine phosphatase activity	4.22E-10	5.27E-07	P99999, P63151, P53041, P36873, P62140, P30153, Q14738, Q08209, P16298, P62136	58	16881	22.91745859	8.26E-08	1.38E-08	127	10	7.8125
GO:0004129~cytochrome-c oxidase activity	1.93E-09	2.41E-06	P13073, P14406, P10606, P14854, P20674, O00483, P00403, P15954	30	16881	35.44566929	3.77E-07	5.39E-08	127	8	6.25
GO:0016887~ATPase activity	1.24E-07	1.55E-04	P24539, P06576, O75964, P48047, Q15257, P30049, P18859, P25705, P56385, O75947, P36542, P56381	183	16881	8.716148186	2.43E-05	3.04E-06	127	12	9.375
GO:0004674~protein serine/threonine kinase activity	1.36E-07	1.70E-04	Q8TDC3, O96013, Q5VT25, Q8N568, Q13177, O15075, Q9Y376, Q7L7X3, Q9UQM7, Q13555, Q7KZI7, Q8IU85, Q13554, Q16513, O75914, Q13557	376	16881	5.656223823	2.67E-05	2.96E-06	127	16	12.5
GO:0005528~FK506 binding	3.03E-07	3.79E-04	Q9NYL4, Q14318, Q02790, P62942, P26885, Q00688	20	16881	39.87637795	5.94E-05	5.94E-06	127	6	4.6875
GO:0008601~protein phosphatase type 2A regulator activity	3.96E-07	4.94E-04	Q13362, P63151, Q15257, P30153, Q14738, Q16537	21	16881	37.97750281	7.75E-05	7.05E-06	127	6	4.6875
GO:0004721~phosphoprotein phosphatase activity	9.85E-07	0.001229959	P53041, P36873, P62140, P67775, Q08209, P16298, P62136	45	16881	20.67664042	1.93E-04	1.61E-05	127	7	5.46875
GO:0019888~protein phosphatase regulator activity	2.20E-06	0.002748865	Q13362, P63151, Q15257, P30153, Q14738, Q16537	29	16881	27.50095031	4.31E-04	3.32E-05	127	6	4.6875
GO:0015078~hydrogen ion transmembrane transporter activity	3.67E-06	0.004578486	P24539, P48201, O75964, P18859, P56385, O75947	32	16881	24.92273622	7.18E-04	5.13E-05	127	6	4.6875
GO:0009055~electron carrier activity	4.88E-06	0.006096156	P99999, P19404, Q9UI09, O75489, P00390, P20674, O75380, O75306	90	16881	11.8152231	9.56E-04	6.38E-05	127	8	6.25
GO:0004683~calmodulin-dependent protein kinase activity	1.95E-05	0.024362414	Q9UQM7, Q13555, Q8IU85, Q13554, Q13557	22	16881	30.20937724	0.003815491	2.39E-04	127	5	3.90625
GO:0005215~transporter activity	2.07E-05	0.025891959	P05141, P06576, P48201, P48047, P30049, P12236, P18859, P55087, P12235, Q9UJS0	202	16881	6.580260388	0.004054583	2.39E-04	127	10	7.8125
GO:0046961~proton-transporting ATPase activity, rotational mechanism	3.90E-05	0.048647672	P06576, P30049, P25705, P36542, P56381	26	16881	25.56178074	0.007605329	4.24E-04	127	5	3.90625
GO:0032403~protein complex binding	1.57E-04	0.196509523	Q96HS1, P63151, P36873, P23284, P51970, O00483, Q16795, P21796, P62136	206	16881	5.807239508	0.030390188	0.001622973	127	9	7.03125
GO:0015207~adenine transmembrane transporter activity	1.65E-04	0.205937917	P05141, P12236, P12235	3	16881	132.9212598	0.031826321	0.001615882	127	3	2.34375
GO:0003735~structural constituent of ribosome	2.62E-04	0.326853045	Q6NUK1, P05141, Q9BV35, O75746, Q6KCM7, P12236, O95182, P12235, Q9UJS0	222	16881	5.388699723	0.050068449	0.00244298	127	9	7.03125
GO:0005516~calmodulin binding	5.43E-04	0.676255472	P54750, Q9UQM7, Q13555, Q08209, Q8IU85, Q13554, Q13557, P16298	189	16881	5.626296713	0.100989861	0.004827445	127	8	6.25
